# Study of Demarcation Line Depth in Transepithelial versus Epithelium-Off Accelerated Cross-Linking (AXL) in Keratoconus

**DOI:** 10.1155/2019/3904565

**Published:** 2019-09-26

**Authors:** Yehia Salah, Kholoud Omar, Ahmed Sherif, Sarah Azzam

**Affiliations:** Department of Ophthalmology, Cairo University, Giza, Egypt

## Abstract

**Aim:**

This is a prospective interventional clinical trial to assess the depth of the demarcation line in transepithelial versus epithelium-off accelerated corneal cross-linking (AXL) in keratoconus patients.

**Methods:**

This prospective clinical trial was conducted on 40 eyes of 20 patients. Each patient had transepithelial AXL in one eye and epithelium-off AXL in the contralateral eye applying UVA light with an irradiance of 45 mW/cm^2^ for 2.4 minutes and 30 mW/cm^2^ for 4 minutes. The depth of the demarcation line was measured using anterior segment OCT (Topcon 3D OCT-2000) one month postoperative for both eyes.

**Results:**

The demarcation line was patchy in 50% of the transepithelial AXL eyes, the other half showing a demarcation line at a mean depth of 183 ± 41.6 *μ*m. In the epithelium-off AXL technique, the demarcation line was well defined in all cases with a mean depth of 219 ± 27.3 *μ*m. There was a statistically significant difference in corneal demarcation line depth between transepithelial and epithelium-off techniques (*P* = 0.008 and *P* < 0.05). The shallower demarcation line in the transepithelial group suggests that it is less effective.

**Conclusion:**

Based on the depth of the demarcation line, the cross-linking effect of epi-off AXL seems more efficacious than after transepithelial AXL. The future will show if the biomechanical effect will be sufficient to stop progression of keratoconus similarly after standard CXL. This trial is registered with NCT04045626.

## 1. Introduction

Keratoconus is a degenerative corneal disorder associated with corneal ectasia and thinning with eventual loss of visual acuity following myopia, irregular astigmatism, and corneal scarring [1]. Corneal collagen cross-linking is a surgical modality for increasing the corneal biomechanical strength to arrest keratoconus progression [2, 3]. Standard corneal cross-linking treatment of progressive keratoconus was introduced by Wollensak et al. from Germany in 2003. It is sometimes also called the Dresden protocol and includes preoperative removal of the central corneal epithelium, application of the photosensitizer riboflavin, and UVA irradiation with an irradiance of 3 mW/cm^2^ for 30 minutes with a total surface dose of 5.4 J/cm^2^ [4]. So-called accelerated cross-linking (AXL) has been proposed to shorten the duration of the procedure. It is based on the Bunsen-Roscoe law of reciprocity increasing the UVA irradiance and significantly reducing the irradiation time resulting in a similar overall UVA dose [5]. Transepithelial cross-linking, gained ground by leaving epithelium intact, improving patient comfort and minimizing the risk of infection associated with epithelial removal. However, its efficacy remained controversial as the epithelial barrier limits the depth and amount of cross-linking, as evidenced by a shallow and uneven distribution of the keratocytes apoptosis on confocal microscopy and ectatic progression in many clinical trials as compared to epithelium-off CXL [6]. The stratified corneal epithelium with tight junctions presents as a lipophilic barrier to the absorption of the hydrophilic riboflavin macromolecule. Standard riboflavin formulations were modified with addition of EDTA, benzalkonium chloride (BAC), and trometamol, which loosen up the epithelial junctions, enhancing the permeability and riboflavin diffusion across the epithelium and superficial stroma [7].

Studies have shown that the effective depth of standard CXL is confined to the anterior 300 um of the cornea as demonstrated by the depth of the keratocyte apoptosis with lacunar edema on histology and the clinical demarcation line [5].

The purpose of our study is to evaluate and compare the depth of the corneal stromal demarcation line using AS-OCT after transepithelial cross-linking versus accelerated epi-off cross-linking procedures.

## 2. Patients and Methods

This prospective comparative interventional study comprised patients with progressive keratoconus. The study was approved by the local ethical committee and followed the principles of the Declaration of Helsinki. 40 eyes of 20 patients were divided into 2 groups: Group A: transepithelial AXL; Group B: epithelium-off AXL. Inclusion criteria were progressive keratoconus, grade 1-2 keratoconus (Amsler–Krumeich classification) Snellen best corrected visual acuity of 0.4 or better, and pachymetry of 400 *μ*m or more at thinnest location.

Progressive keratoconus was defined as an increase in *k*_max_ of 1.00 diopter [D], an increase of manifest refraction cylinder of 1.0 D, or an increase of manifest refraction spherical equivalent of 0.5 D over the period of 1 year. CXL treatment was performed with a KXL I UVA source (Avedro Inc., Waltham, MS, USA).

In transepithelial AXL, ParaCel™ (containing 0.25% riboflavin and benzalkonium chloride) was instilled in the form of 1 drop every 1.5 minutes for 4.5 minutes, and then VibeX Xtra was instilled in the form of 4 drops at 5.5 minutes followed by 1 drop at 6.5 minutes and again every 1.5 minutes for three times. Total soak time of riboflavin was 11 minutes followed by UVA irradiation with an intended irradiance of 45 mW/cm^2^ (total surface dose of 7.2 J/cm^2^) for 2.4 minutes.

In epithelium-off AXL, the corneal epithelium was removed using a blunt spatula (8.0 to 9.0 mm in diameter). VibeX Rapid (riboflavin 0.1%) was instilled on the center of the cornea every 2 minutes for 10 minutes followed by ultraviolet-A irradiation with an intended irradiance of 30 mW/cm^2^ (total surface dose of 7.2 J/cm^2^) for 4 minutes. At the end of the procedure, a bandage contact lens was placed over the cornea and removed after full epithelization (postoperative 4-5 days). Postoperative medications included moxifloxacin 0.5% (Vigamox, Alcon, Inc., Canada) 4 times daily for 2 weeks and artificial tears 4 times daily for one month with ascorbic acid for one month. After removal of contact lens, patients received fluoromethelone acetate 4 times daily tapered over 2 weeks.

### 2.1. Evaluation of Depth of the Demarcation Line

Anterior segment optical coherence tomography (AS-OCT) (Topcon 3D OCT-2000) was performed to evaluate the corneal stroma for the presence of the demarcation line 1 month postoperative by an independent observer unaware of the purpose of this study. The demarcation line measurements of this study include the epithelial thickness of about 50 *μ*m.

## 3. Statistical Analysis

Microsoft EXCEL 2010 and SPSS of Windows software (version 20.0, SPSS Inc.) were used for statistical analysis. Normality of all data samples was first checked by means of the Kolmogorov–Smirnov test. Then *t*-test was possible, and the Student *t*-test for paired data was performed for all parameter comparisons between preoperative and postoperative examinations using the same level of significance (*P* < 0.05) in all cases. And in comparing the 2 groups we used the unpaired *t*-test.

## 4. Results

This clinical prospective study included 40 eyes of 20 patients; each patient had transepithelial CXL in one eye and epithelium-off CXL in the other. 11 females and 9 males were included in the study. The mean patient age was 32 (ranging from 16 to 40 years). The mean preoperative CCT was 484.55 ± 35.47 *μ* in the transepithelial group and 477.75 ± 37.06 *μ* in the epi-off group (*P*=0.557).

### 4.1. Demarcation Line One Month Postoperatively

**Figure 1:**
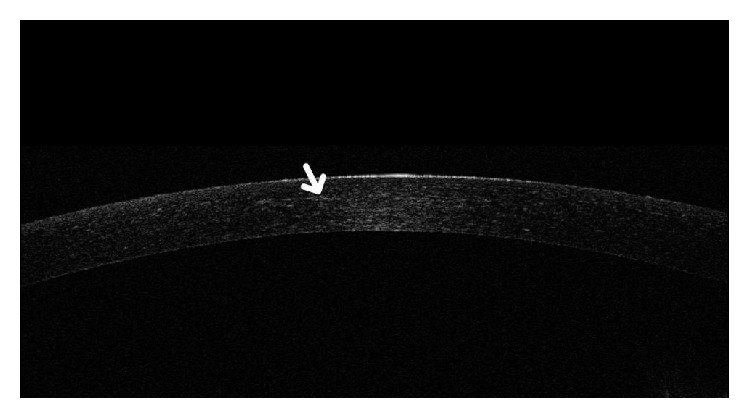
A patchy corneal demarcation line (white arrow) after transepithelial cross-linking.

The demarcation line was patchy and ill-defined in 50% of the transepithelial eyes, the depth of which could not be assessed (Figure 1). The other half showed a clear demarcation line. However, there was a clear demarcation line in all epithelium-off eyes. There was a statistically significant difference in the incidence of a patchy corneal demarcation line between transepithelial and epithelium-off techniques (*P* = 0.001 and *P* < 0.05).

### 4.2. Demarcation Line Depth One Month Postoperatively

**Figure 2:**
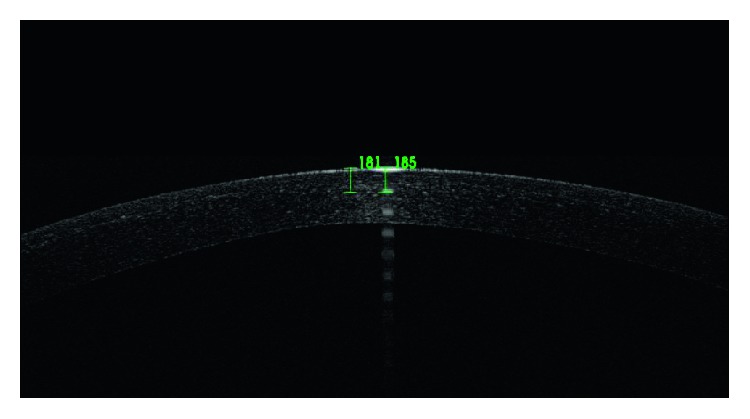
A barely visible corneal demarcation line at a central depth of 185 *μ*m after transepithelial cross-linking.

**Figure 3:**
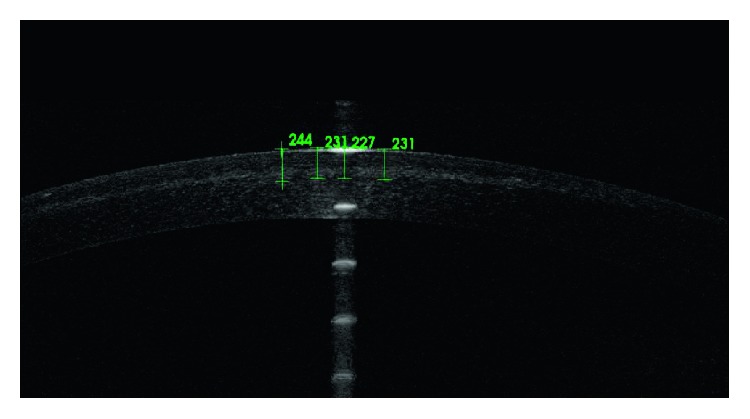
A corneal demarcation line at a central depth of 227 *μ*m after epithelium-off cross-linking.

The mean depth of the corneal demarcation line was 183 *μ*m in the transepithelial technique and 219 *μ*m in the epithelium-off technique (Figures 2 and 3). There was a statistically significant difference in corneal demarcation line depth between transepithelial and epithelium-off techniques (*P* = 0.008 and *P* < 0.05; Table 1).

### 4.3. Postoperative Complications

Grade I corneal haze (graded according to Fantes scoring) was found in 4 eyes only in transepithelial CXL and in 9 eyes in epithelium-off CXL. No delayed epithelialization or infective keratitis was seen in either group.

## 5. Discussion

Corneal CXL with riboflavin and ultraviolet-A (UVA) is a technique to strengthen corneal tissue [8]. The corneal epithelium is the critical obstacle to riboflavin permeation into the corneal stroma [8, 9]. However, the removal of epithelium can cause some complications such as severe postoperative pain, temporary visual blurring, epithelial healing problems, haze, viral reactivation, and even corneal melting. To prevent these serious problems, some researchers have performed a number of studies on a special riboflavin solution, which can penetrate the intact epithelium, such as benzalkonium chloride, EDTA, and trometamol [7].

In the epi-off group, our results were comparable to Moramarco et al. [10] and Jiang et al. [11] who performed epi-off AXL using pulsed UVA light with 8 minutes of UVA exposure at 30 mW/cm^2^ and energy dose of 7.2 J/cm^2^. The mean depth of the stromal demarcation line was 213 ± 47.38 *μ*m and 201.64 ± 27.72 *μ*m, respectively.

Bouheraoua et al. showed similar results using irradiance of 30 mW/cm^2^ for 3 minutes (5.4 J/cm^2^ surface dose). The corneal demarcation line was visible in 87.5% (mean depth 184.2 ± 38.9 *μ*m) in the A-CXL group [12].

The depth of the demarcation line in our study of epithelium-off cases was found to be shallower (mean 219 *μ*) than in other studies using different accelerated protocols. In a study by Kymionis et al., patients were treated for 10 minutes in an intended irradiance of 9.0 mW/cm^2^ corresponding to a total surface dose of 5.4 J/cm^2^. The mean depth of the corneal stromal demarcation line was 288.46 ± 42.37 mm [13]. Another study by Kymionis et al., using high intensity CXL protocol of 18 mW/cm^2^ UVA irradiation for 7 minutes, the mean corneal stromal demarcation line depth was 313.37 ± 48.85 um [14].

Similarly, Lhuillier et al. using an accelerated protocol of 10 min, 9 mW/cm^2^ ultraviolet-A, corresponding to a total surface dose of 5.4 J/cm^2^ reported a mean depth of the stromal demarcation line of 331.2 ± 62.7 *μ*m [15].

Our results on AXL were markedly different from Yam et al. who used a classic protocol of 3.0 mW/cm^2^ for 30 minutes (surface dose 5.4 J/cm^2^). The median CXL demarcation line depth at the central cornea was 323.0 ± 48.6, suggesting a significantly weaker cross-linking effect of AXL compared to standard CXL [5].

As regards for the transepithelial AXL technique, UVA irradiation was performed for 2.4 minutes, with an intended power of 45 mW/cm^2^, giving a total energy of 7.2 J/cm^2^. 50% of the cases had a patchy demarcation line and 50% had a demarcation line at a mean depth of 183 *μ*m.

These results were similar to Artola et al. who reported a demarcation line depth between 153 and 230 *μ*m, with a mean value of 205.19 *μ*m [16].

Our study's findings support the review by Liu et al. who collected eligible studies retrieved from four electronic databases, including CENTRAL, ClinicalTrials.gov, PupMed, and Ovid MEDLINE. 24 comparative studies on transepithelial cross-linking compared with standard cross-linking were included and pooled for analysis. The demarcation line of standard CXL was significantly deeper than that of transepithelial cross-linking; the mean difference of the depth of the demarcation line (DDL) was 133.49 *μ*m [17].

Both transepithelial and epi-off AXL have been shown to be biomechanically less efficacious than standard CXL [5]. The weaker effect is also reflected by the present study with the difference in the DL depth and the often patchy and irregular configuration or even absence of the DL in transepithelial AXL probably due to an inhomogenous distribution of the photosensitizer riboflavin [18].

In conclusion, we found that the demarcation line was not consistent in transepithelial cases, and it was found to be more superficial than in standard CXL as has also been found by other studies with different AXL protocols. A shallower demarcation line demonstrates a weaker cross-linking effect. It has been shown that the CXL effect has a natural saturation effect which is maximally high in a specific tissue layer. The effect can only be increased further by increasing the volume of cross-linked tissue which is reflected and revealed by the greater depth of DL [5].

The future will show if the clearly weaker cross-linking effect of epi-off and transepithelial AXL compared to standard CXL will be sufficient to stop progression of keratectasia.

## Figures and Tables

**Table 1:** Summary of results.

	Transepithelial, mean	SD	Accelerated epithelium-off, mean	SD	*P* value
Central corneal thickness	484.55	35.47	477.75	37.06	0.557
Demarcation line depth	183.0	41.60	219.30	27.30	0.008

## Data Availability

The data used to support the findings of this study are available from the corresponding author upon request.
